# Time Fractional Fisher–KPP and Fitzhugh–Nagumo Equations

**DOI:** 10.3390/e22091035

**Published:** 2020-09-16

**Authors:** Christopher N. Angstmann, Bruce I. Henry

**Affiliations:** School of Mathematics and Statistics, UNSW, Sydney 2052 NSW, Australia

**Keywords:** fractional diffusion, continuous time random walks, reaction–diffusion equations, reaction kinetics

## Abstract

A standard reaction–diffusion equation consists of two additive terms, a diffusion term and a reaction rate term. The latter term is obtained directly from a reaction rate equation which is itself derived from known reaction kinetics, together with modelling assumptions such as the law of mass action for well-mixed systems. In formulating a reaction–subdiffusion equation, it is not sufficient to know the reaction rate equation. It is also necessary to know details of the reaction kinetics, even in well-mixed systems where reactions are not diffusion limited. This is because, at a fundamental level, birth and death processes need to be dealt with differently in subdiffusive environments. While there has been some discussion of this in the published literature, few examples have been provided, and there are still very many papers being published with Caputo fractional time derivatives simply replacing first order time derivatives in reaction–diffusion equations. In this paper, we formulate clear examples of reaction–subdiffusion systems, based on; equal birth and death rate dynamics, Fisher–Kolmogorov, Petrovsky and Piskunov (Fisher–KPP) equation dynamics, and Fitzhugh–Nagumo equation dynamics. These examples illustrate how to incorporate considerations of reaction kinetics into fractional reaction–diffusion equations. We also show how the dynamics of a system with birth rates and death rates cancelling, in an otherwise subdiffusive environment, are governed by a mass-conserving tempered time fractional diffusion equation that is subdiffusive for short times but standard diffusion for long times.

## 1. Introduction

Reaction–diffusion partial differential equations are among the most widely used equations in applied mathematics modelling. These equations govern the time evolution of concentrations, or population densities, of species, at different spatial locations, that are diffusing and reacting. Applications include the spatio-temporal spread of epidemics, the spatial spread of invasive species and the development of animal coat patterns [1,2,3]. In these modelling equations, diffusion is represented by a spatial Laplacian operating on the population densities, and reactions are included as additive terms representing changes per unit time in population densities through reaction rates. In well-mixed systems the reaction rate equations can often be derived from the law of mass-action [4]. A famous example of a reaction–diffusion equation is the Fisher–KPP equation named after Fisher [5] and Kolmogorov, Petrovsky and Piskunov [6]. The standard reaction–diffusion representation of this equation is

(1) $$\frac{\partial u(x,t)}{\partial t}=D\frac{\partial^{2}u\left(x,t\right)}{\partial x^{2}}+ru\left(x,t\right)\left(1-u\left(x,t\right)\right),\;D>0,r>0.$$

Here, u(x,t) represents the population density of a species, $D\frac{\partial^{2}u\left(x,t\right)}{\partial x^{2}}$ represents the diffusion of the species and ru(x,t)(1−u(x,t)) represents the reactions of the species. In the absence of diffusion, the time rate of change in the population density is the same at all points in space and is given by

(2) $$\frac{\partial u(x,t)}{\partial t}=ru\left(x,t\right)\left(1-u\left(x,t\right)\right).$$

In this example and in the following, for simplicity, we have considered systems in one spatial dimension. Extensions to higher spatial dimensions are possible.

Over the past two decades, there has been a growing awareness of fractional diffusion, where diffusion cannot be modelled using a standard Laplacian and the mean square displacement of diffusing species does not grow linearly in time, as anticipated by Einstein’s famous modelling of Brownian motion [7]. In particular, following widespread observations in biological systems, there has been a great deal of attention focussed on fractional subdiffusion, characterized by the mean square displacement of a population spreading as a sublinear power law in time. It is now generally accepted that if subdiffusion arises from particles being trapped for arbitrarily long periods of time, the appropriate equation to model subdiffusion is the time fractional diffusion equation [8]

(3) $$\frac{\partial u(x,t)}{\partial t}={\;}_{0}D_{t}^{1-\gamma }\frac{\partial^{2}u\left(x,t\right)}{\partial x^{2}},\;0<\gamma <1,$$

which can be derived [9,10] from a continuous time random walk (CTRW) [11] with a power law waiting time density. In this equation,

(4) $${\;}_{0}D_{t}^{1-\gamma }y\left(x,t\right)=\frac{1}{\Gamma (\gamma )}\frac{\partial }{\partial t}\int_{0}^{t}\frac{y(x,t^{\prime })}{{\left(t-t^{\prime }\right)}^{1-\gamma }}\;dt^{\prime }$$

is the Riemann–Liouville fractional derivative of order 1−γ, see, for example, reference [12]. It might be anticipated that the appropriate evolution equation to model subdiffusion, with reactions governed by the reaction rate equation,

(5) $$\frac{\partial u(x,t)}{\partial t}=f\left(u\left(x,t\right)\right),$$

would be

(6) $$\frac{\partial u(x,t)}{\partial t}={\;}_{0}D_{t}^{1-\gamma }\frac{\partial^{2}u\left(x,t\right)}{\partial x^{2}}+f\left(u\left(x,t\right)\right).$$

Indeed, such an equation had been derived from an underyling CTRW model, under certain assumptions, [13], however it is not valid in general. For example, the simple model equation

(7) $$\frac{\partial u(x,t)}{\partial t}={\;}_{0}D_{t}^{1-\gamma }\frac{\partial^{2}u\left(x,t\right)}{\partial x^{2}}-u\left(x,t\right),$$

can have unphysical negative solutions [14].

The time fractional subdiffusion equation is also often written as [15]

(8) $$\frac{\partial^{\gamma }u\left(x,t\right)}{\partial t^{\gamma }}=\frac{\partial^{2}u\left(x,t\right)}{\partial x^{2}},\;0<\gamma <1,$$

where

(9) $$\frac{\partial^{\gamma }}{\partial t^{\gamma }}y\left(x,t\right)=\frac{1}{\Gamma (1-\gamma )}\int_{0}^{t}\frac{\frac{\partial }{\partial t}y\left(x,t^{\prime }\right)}{{\left(t-t^{\prime }\right)}^{\gamma }}\;dt^{\prime }$$

denotes a Caputo fractional derivative, see, for example, reference [12]. There has been quite a bit written in the published literature on the greater physical practicality of the Caputo derivative over the Riemann–Liouville derivative, but this is largely unfounded [12]. Note, however, that if one takes Equation (8) as the starting evolution equation for subdiffusion then this is suggestive of the following reaction–subdiffusion equation,

(10) $$\frac{\partial^{\gamma }u\left(x,t\right)}{\partial t^{\gamma }}=\frac{\partial^{2}u\left(x,t\right)}{\partial x^{2}}+f\left(u\left(x,t\right)\right).$$

Equations along the lines of Equation (10) are particularly widespread in the literature with the motivation that fractional derivatives incorporate a history dependence, and solutions of Equation (10) remain positive. Equation (10) can be derived from a CTRW where particles are being removed or added instantaneously at the start of the waiting times between jumps, but only under the contrived constraint that $\frac{\partial^{1-\gamma }f\left(u\left(x,t\right)\right)}{\partial t^{1-\gamma }}$ represents the cumulative total of additions and removals to the arrival density of particles at position *x* and time *t* [14].

The derivation of reaction–subdiffusion equations from physically consistent CTRWs has been carried out in a series of papers [14,16,17,18,19,20,21,22,23,24,25,26]. The main lessons from this body of work are: (i) The governing equations are different depending on whether or not new born particles inherit the waiting times of their parents. (ii) Birth terms and death terms must be treated differently. (iii) In the case where particles are removed, but not instantaneously at the start of the waiting time between jumps, the reaction and subdiffusion terms are not additive. The following equation [21,24],

(11) $$\begin{array}{ccc} \frac{\partial u(x,t)}{\partial t} & = & D_{\gamma }\frac{\partial^{2}}{\partial x^{2}}\left[e^{-\int_{0}^{t}a\left(u\left(x,t^{\prime }\right),x,t^{\prime }\right)\;dt^{\prime }}{\;}_{0}D_{t}^{1-\gamma }\left(e^{\int_{0}^{t}a\left(u\left(x,t^{\prime }\right),x,t^{\prime }\right)\;dt^{\prime }}u\left(x,t\right)\right)\right]\\ & & +c(u(x,t),x,t)-a(u(x,t),x,t)u(x,t), \end{array}$$

which was derived from a continuous time random walk model, provides the evolution equation for particles undergoing subdiffusion with particles annihilated at a per capita rate, a(u(x,t,x,t) and created at a rate c(u(x,t),x,t). In the derivation of this equation it was assumed that newborn particles do not inherit the waiting times of their parents.

In the remainder of this paper we explore examples related to Equation (11). These examples have been selected to emphasize the importance of considering the details of the reaction kinetics when dealing with reaction–subdiffusion problems. Whilst there have been many papers published on various methods of solution for variants of Equation (10) (see, for example, [27,28,29,30,31]), there have been very few papers published considering algebraic or numerical solution methods for variants of Equation (11). We hope that the examples below will stimulate further activity in this area, where the physical motivation for the modelling equation is stronger.

## 2. Examples

### 2.1. Birth and Death Balance

As a first example, we consider a population density of u(x,t) particles per unit volume that are diffusing with a per capita death rate α and a birth rate αu(x,t). The reaction rate equation reflecting this balance between births and deaths, in a well-mixed population, at a location *x* is

(12) $$\frac{\partial u(x,t)}{dt}=0,$$

and thus the standard reaction–diffusion equation describing this system is

(13) $$\frac{\partial u(x,t)}{dt}=D\frac{\partial^{2}u\left(x,t\right)}{\partial x^{2}}.$$

The simple generalization of this equation for subdiffusive transport is

(14) $$\begin{array}{ccc} \frac{\partial u(x,t)}{dt} & = & D_{\gamma }{\;}_{0}D_{t}^{1-\gamma }\frac{\partial^{2}u\left(x,t\right)}{\partial x^{2}}\\ \; & = & D_{\gamma }\frac{\partial^{2}}{\partial x^{2}}\left[{\;}_{0}D_{t}^{1-\gamma }u\left(x,t\right)\right]. \end{array}$$

Indeed, if there were no births or deaths then the reaction rate equation would still be given by Equation (12); and Equation (14) is the appropriate equation to describe subdiffusion without births or deaths. However, the reaction–subdiffusion equation, following Equation (11), and using the reaction rate kinetics a(u(x,t),x,t)=α and c(u(x,t),x,t)=αu(x,t), which are also consistent with the rate equation, Equation (12), is remarkably different;

(15) $$\frac{\partial u(x,t)}{\partial t}=D_{\gamma }\frac{\partial^{2}}{\partial x^{2}}\left[e^{-\alpha t}{\;}_{0}D_{t}^{1-\gamma }\left(e^{\alpha t}u\left(x,t\right)\right)\right],\;\alpha >0.$$

The fundamental difference between Equations (14) and (15) is that in the former equation the Laplacian operates on a time fractional derivative and in the latter the Laplacian operates on a tempered time fractional derivative [32,33]. In the more general time fractional reaction–diffusion equation, Equation (11), the term in brackets following the Laplacian defines a generalized tempered time fractional derivative. The physical interpretation of the tempering is that if particles are being annihilated at a given rate while they wait then they cannot wait an arbitrarily long time at a given location. Note that both Equations (14) and (15) are mass conserving and thus Equation (15) then defines a mass conserving, tempered, time fractional diffusion equation.

The mean square displacement of the diffusing particles, $\langle x^{2}\left(t\right)\rangle$, provides a clear measurable difference between particles following Equation (14) or Equation (15). In the former case, identified as $\langle x_{I}^{2}\left(t\right)\rangle$, we have [8],

(16) $$\langle x_{I}^{2}\left(t\right)\rangle =\frac{2D_{\gamma }}{\Gamma (1+\gamma )}t^{\gamma },$$

and in the latter case, identified as $\langle x_{II}^{2}\left(t\right)\rangle$, we have (Appendix A)

(17) $$\langle x_{II}^{2}\left(t\right)\rangle =2D_{\gamma }e^{-\alpha t}t^{\gamma }E_{1,\gamma }^{\left(1\right)}\left(\alpha t\right),$$

where

(18) $$E_{1,\gamma }^{\left(1\right)}\left(z\right)=\frac{d}{dz}\sum_{k=0}^{\infty }\frac{z^{k}}{\Gamma (\gamma +k)}$$

is the derivative of a generalized Mittag–Leffler function [34]. Note that at short times,

(19) $$\langle x_{II}^{2}\left(t\right)\rangle ∼\frac{2D_{\gamma }}{\Gamma (1+\gamma )}t^{\gamma },$$

but at large times, using the asymptotic expansion of the generalized Mittag–Leffler function (Equation (6) in [35]),

(20) $$\langle x_{II}^{2}\left(t\right)\rangle ∼2D_{\gamma }\alpha^{1-\gamma }t.$$

Thus, mass conserving tempered time fractional diffusion is not anomalous at long times.

We can also write down explicit expressions for solutions to Equations (14) and (15), labelled as $u_{I}\left(x,t\right)$ and $u_{II}\left(x,t\right)$, respectively. For simplicity we consider the infinite domain Greens function solutions with initial condition u(x,0)=δ(x).

The Greens function solution of the fractional diffusion equation Equation (14) can be written as [8]

(21) $$u_{I}\left(x,t\right)=\frac{1}{\sqrt{4\pi D_{\gamma }t^{\gamma }}}H_{1,2}^{2,0}\left[\frac{x^{2}}{4D_{\gamma }t^{\gamma }}\left|\begin{array}{cc} \left(1-\frac{\gamma }{2},\gamma \right) & \\ \left(0,1\right) & \left(\frac{1}{2},1\right) \end{array}\right.\right],$$

where *H* denotes a Fox H-function [36], see Equation (A11).

To find the Greens function solution $u_{II}\left(x,t\right)$ we first note that Equation (15) can be re-written as

(22) $$\frac{\partial v(x,t)}{\partial t}=D_{\gamma }\frac{\partial^{2}}{\partial x^{2}}{\;}_{0}D_{t}^{1-\gamma }v\left(x,t\right)+\alpha v\left(x,t\right),$$

where

(23) $$v\left(x,t\right)=e^{\alpha t}u_{II}\left(x,t\right).$$

The Greens function solution of Equation (22) can be obtained as a special case of the more general results in Appendix B of [14], yielding

(24) $$v\left(x,t\right)=\frac{1}{\sqrt{4\pi D_{\gamma }t^{\gamma }}}\sum_{j=0}^{\infty }\frac{{\left(\alpha t\right)}^{j}}{j!}H_{1,2}^{2,0}\left[\frac{x^{2}}{4D_{\gamma }t^{\gamma }}\left|\begin{array}{cc} \left(1-\frac{\gamma }{2}+j,\gamma \right) & \\ \left(0,1\right) & \left(\frac{1}{2}+j,1\right) \end{array}\right.\right],$$

and then using Equation (23) we have

(25) $$u_{II}\left(x,t\right)=e^{-\alpha t}\frac{1}{\sqrt{4\pi D_{\gamma }t^{\gamma }}}\sum_{j=0}^{\infty }\frac{{\left(\alpha t\right)}^{j}}{j!}H_{1,2}^{2,0}\left[\frac{x^{2}}{4D_{\gamma }t^{\gamma }}\left|\begin{array}{cc} \left(1-\frac{\gamma }{2}+j,\gamma \right) & \\ \left(0,1\right) & \left(\frac{1}{2}+j,1\right) \end{array}\right.\right].$$

In Appendix B, we show that the Fox functions in Equations (21) and (25) can be simplified for $\gamma =\frac{1}{2}$ in terms of Miejer G-Functions [37], see Equation (A12), which have the advantage that they can readily be evaluated using computer algebra packages such as MATHEMATICA and MAPLE. Using the result of Equation (A19) from the Appendix B, we can write (see also [8] in the case of $u_{I}\left(x,t\right)$)

(26) $$u_{I}\left(x,t\right)=\frac{1}{\sqrt{8\pi^{3}Dt^{\frac{1}{2}}}}G_{0,3}^{3,0}\left[{\left(\frac{x^{2}}{16Dt^{\frac{1}{2}}}\right)}^{2}\left|\begin{array}{c} -\\ 0,\frac{1}{4},\frac{1}{2} \end{array}\right.\right],$$

and

(27) $$u_{II}\left(x,t\right)=e^{-\alpha t}\frac{1}{\sqrt{8\pi^{3}Dt^{\frac{1}{2}}}}\sum_{j=0}^{\infty }\frac{{\left(2\alpha t\right)}^{j}}{j!}G_{1,4}^{4,0}\left[{\left(\frac{x^{2}}{16Dt^{\frac{1}{2}}}\right)}^{2}\left|\begin{array}{c} \frac{3}{4}+j\\ 0,\frac{1}{2},\frac{1}{4}+\frac{j}{2},\frac{3}{4}+\frac{j}{2} \end{array}\right.\right].$$

**Figure 1:**
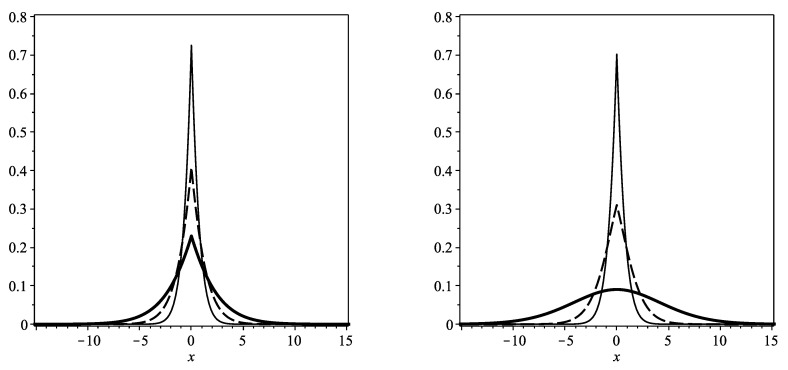
Plots of Equation (26), the algebraic solution to Equation (14), (**left**), and Equation (27), the algebraic solution to Equation (15), (**right**), at times t=0.1 (solid line), t=1.0 (dashed line) and t=10.0 (bold solid line). The reaction parameter α=1, and the fractional order derivative is taken to be γ=0.5 in each of these plots.

Note that the expression for $u_{II}\left(x,t\right)$ simplifies to the expression for $u_{I}\left(x,t\right)$ if α=0. If $\left|x\right|\gg 4\sqrt{Dt^{\frac{1}{2}}}$ then we can use asymptotic expansions for $G_{0,3}^{3,0}\left(z\right)$ and $G_{1,4}^{4,0}\left(z\right)$ with z≫1 (see Appendix B) to write

(28) $$u_{I}\left(x,t\right)∼\frac{1}{\sqrt{8\pi^{3}Dt^{\frac{1}{2}}}}\exp\left(-3{\left(\frac{x^{2}}{16Dt^{\frac{1}{2}}}\right)}^{\frac{2}{3}}\right){\left(\frac{x^{2}}{16Dt^{\frac{1}{2}}}\right)}^{\frac{1}{2}}\frac{M_{0}}{{\left(\frac{x^{2}}{16Dt^{\frac{1}{2}}}\right)}^{\frac{2}{3}}-1},$$

and

(29) $$u_{II}\left(x,t\right)∼Me^{\alpha t}u_{I}\left(x,t\right),$$

where $M_{0}$ and *M* are constant terms. The solutions $u_{I}\left(t\right)$, Equation (26), and $u_{II}\left(t\right)$, Equation (27) are plotted in Figure 1, with α=1 and D=1, at times t=0.1, t=1.0 and t=10.0. The solutions are very similar at early times but the corner at the origin, which is characteristic of subdiffusion, is less sharp at longer times in the solution of Equation (27).

The lesson from this simple example is that reaction dynamics equations do not contain sufficient information on their own to provide model equations for reaction–subdiffusion systems even in well-mixed systems. In the case of standard diffusion, the evolution of the population density is only affected by the overall reaction rates, in a well-mixed system, but not the details of the reaction kinetics. In a standard reaction–diffusion system, the dynamics with no births and no deaths is the same as if there were births and deaths but the rates cancelled out. The reaction–diffusion equation with these reaction kinetics has no memory of the birth and death processes. This is very different in the case of subdiffusion where the details of the reaction kinetics are important to the overall dynamics of the system. The subdiffusive system retains a memory that there were particles that were created and annihilated. Moreover, the particle deaths temper the fractional diffusion. The example in the next section further highlights the significance of the reaction kinetics in reaction–subdiffusion systems.

### 2.2. Fractional Fisher–KPP Equation

The reaction rate equation for the Fisher–KPP Equation (1) is given in Equation (2). There are many different reaction kinetics that could be considered that are consistent with Equation (2). For example, the term (1−u(x,t)) in its entirety could represent a per capita birth rate if is is strictly positive, or a per capita death rate if it is strictly negative. This term could also be regarded as being composed of two terms, a constant per capita birth term and a linear per capita death term. These three possibilities are highlighted for illustrative purposes below to show how different subdiffusion–reaction equations apply depending on the reaction kinetics.

(i) Constant per capita birth rate, c(u(x,t),x,t)=ru(x,t), linear per capita death rate, a(u(x,t),x,t)=ru(x,t),
  (30) $$\begin{array}{ccc} \frac{\partial u(x,t)}{\partial t} & = & D_{\gamma }\frac{\partial^{2}}{\partial x^{2}}\left[e^{-\int_{0}^{t}ru\left(x,t^{\prime }\right)\;dt^{\prime }}{\;}_{0}D_{t}^{1-\gamma }\left(e^{\int_{0}^{t}ru\left(x,t^{\prime }\right)\;dt^{\prime }}u\left(x,t\right)\right)\right]\\ & & +ru(x,t)(1-u(x,t)),\;u(x,t)\geq 0 \end{array}$$
(ii) No births, c(u(x,t),x,t)=0, linear per capita death rate, a(u(x,t),x,t)=r(1−u(x,t)),
  (31) $$\begin{array}{ccc} \frac{\partial u(x,t)}{\partial t} & = & D_{\gamma }\frac{\partial^{2}}{\partial x^{2}}\left[e^{-\int_{0}^{t}r\left(1-u\left(x,t^{\prime }\right)\right)\;dt^{\prime }}{\;}_{0}D_{t}^{1-\gamma }\left(e^{\int_{0}^{t}r\left(1-u\left(x,t^{\prime }\right)\right)\;dt^{\prime }}u\left(x,t\right)\right)\right]\\ & & +r(1-u(x,t))u(x,t),\;u(x,t)\geq 1. \end{array}$$
(iii) Linear per capita birth rate, c(u(x,t),x,t)=ru(x,t)(1−u(x,t)), no deaths, a(u(x,t),x,t)=0,
  (32) $$\frac{\partial u(x,t)}{\partial t}=D_{\gamma }\frac{\partial^{2}}{\partial x^{2}}\left[{\;}_{0}D_{t}^{1-\gamma }u\left(x,t\right)\right]+ru\left(x,t\right)\left(1-u\left(x,t\right)\right),\;0\leq u\left(x,t\right)<\leq 1.$$

Note that none of the factional Fisher–KPP reaction–diffusion equations can be expressed in the form

(33) $$\frac{\partial^{\gamma }u\left(x,t\right)}{\partial t^{\gamma }}=D_{\gamma }\frac{\partial^{2}u\left(x,t\right)}{\partial x^{2}}+ru\left(x,t\right)\left(1-u\left(x,t\right)\right),$$

which results from simply replacing the integer order time derivative with a fractional order Caputo derivative. As noted above, an equation of this form could only be obtained from a CTRW if $\frac{\partial^{1-\gamma }}{\partial t^{1-\gamma }}\left(ru(x,t)(1-u(x,t)\right)$ is contrived as the cumulative instantaneous creation and annihilation of particles at the start of the waiting time between particle jumps at position *x* and time *t* [14].

The Greens function solutions for the nonlinear fractional reaction–diffusion equations, Equations (30)–(32), cannot be obtained simply using Fourier–Laplace transform methods. However, it is possible to find numerical solutions using the discrete time random walk methods described in [38].

The Fisher–KPP reaction rate equation, Equation (2) can be motivated by different chemical reactions consistent with the law of mass action [4]. One possibility is that of a single species *A* which undergoes coalescence reactions $A+A\overset{r}{\to }A$, and degradation reactions $A\overset{r}{\to }A+A$; also referrred to as reversible coagulation dynamics [39]. In this scenario the creation term, ru(x,t), arises from degradation and the annihilation term, $-r(u^{2}\left(x,t\right)$, arises from coalescence. Another possibility is a branching–coalescence scheme [17], B+X⇌X+X, with the concentration of *B* maintained at a constant level. Equation (30) is a fractional Fisher–KPP reaction–diffusion equation consistent with each of the reaction schemes described here and it was obtained earlier for the branching–coalescence reaction scheme in [17].

### 2.3. Fractional Fitzhugh–Nagumo Equation

A widely studied reaction–diffusion system used to model wave propagation and pattern formation in excitable media is the Fitzhugh–Nagumo system of equations [40,41]

(34) $$\begin{array}{ccc} \frac{\partial v(x,t)}{\partial t} & = & D_{v}\frac{\partial^{2}v\left(x,t\right)}{\partial x^{2}}+v\left(x,t\right)\left(v\left(x,t\right)-a\right)\left(1-v\left(x,t\right)\right)-w\left(x,t\right),\;D_{v}\geq 0,a\geq 0 \end{array}$$

(35) $$\begin{array}{ccc} \frac{\partial w(x,t)}{\partial t} & = & D_{w}\frac{\partial^{2}w\left(x,t\right)}{\partial x^{2}}+\epsilon \left(v(x,t)-bw(x,t)\right)\;D_{w}\geq 0,\epsilon \geq 0,b\geq 0, \end{array}$$

named after Fizthugh [42] and Nagumo [43]. In recent years, the single component fractional equation

(36) $$\frac{\partial^{\alpha }u\left(x,t\right)}{\partial t^{\alpha }}=D_{u}\frac{\partial^{2}u\left(x,t\right)}{\partial x^{2}}+u\left(x,t\right)\left(u\left(x,t\right)-a\right)\left(1-u\left(x,t\right)\right)$$

has been studied as a test equation for various methods of solution of time fractional reaction–diffusion equations (see, for example, [27,30] and references there-in).

A time fractional Fitzhugh–Nagumo system of equations consistent with Equation (11), derived from a CTRW formalism, can be obtained by identifying per capita annihilation rates, $a_{v}$ and $a_{w}$, and creation rates, $c_{v}$ and $c_{w}$, as follows:

(37) $$\begin{array}{ccc} a_{v}\left(v\left(x,t\right),w\left(x,t\right)\right) & = & a+v^{2}\left(x,t\right)+\frac{w(x,t)}{v(x,t)}, \end{array}$$

(38) $$\begin{array}{ccc} c_{v}\left(v\left(x,t\right),w\left(x,t\right)\right) & = & \left(1+a\right)v^{2}\left(x,t\right), \end{array}$$

(39) $$\begin{array}{ccc} a_{w}\left(v\left(x,t\right),w\left(x,t\right)\right) & = & \epsilon b, \end{array}$$

(40) $$\begin{array}{ccc} c_{w}\left(v\left(x,t\right),w\left(x,t\right)\right) & = & \epsilon v(x,t). \end{array}$$

The corresponding time fractional Fitzhugh–Nagumo system is given by

$$\begin{array}{ccc} \frac{\partial v(x,t)}{\partial t} & = & D_{v,\gamma }\frac{\partial^{2}}{\partial x^{2}}\left[e^{-\int_{0}^{t}\left(v^{2}\left(x,t^{\prime }\right)+a+w\left(x,t^{\prime }\right)\right)\;dt^{\prime }}{\;}_{0}D_{t}^{1-\gamma }\left(e^{\int_{0}^{t}\left(v^{2}\left(x,t^{\prime }\right)+a+w\left(x,t^{\prime }\right)\right)\;dt^{\prime }}v\left(x,t\right)\right)\right] \end{array}$$

(41) $$\begin{array}{ccc} & & +v(x,t)(v(x,t)-a)(1-v(x,t)-w(x,t), \end{array}$$

(42) $$\begin{array}{ccc} \frac{\partial w(x,t)}{\partial t} & = & D_{w,\gamma }\frac{\partial^{2}}{\partial x^{2}}\left[e^{-\epsilon bt}{\;}_{0}D_{t}^{1-\gamma }\left(e^{\epsilon bt}w\left(x,t\right)\right)\right]+\epsilon v\left(x,t\right)-\epsilon bw\left(x,t\right). \end{array}$$

If w(x,t)=0 this identifies a single component time fractional equation

(43) $$\begin{array}{ccc} \frac{\partial u(x,t)}{\partial t} & = & D_{\gamma }\frac{\partial^{2}}{\partial x^{2}}\left[e^{-\int_{0}^{t}\left(u^{2}\left(x,t^{\prime }\right)+a\right)\;dt^{\prime }}{\;}_{0}D_{t}^{1-\gamma }\left(e^{\int_{0}^{t}\left(u^{2}\left(x,t^{\prime }\right)+a\right)\;dt^{\prime }}u\left(x,t\right)\right)\right]\\ & & +u(x,t)(u(x,t)-a)(1-u(x,t), \end{array}$$

which could be called a time fractional Fitzhugh–Nagumo equation, although the nomenclature could be misleading because a single component equation, without external sources or sinks, could not display Fitzhugh–Nagumo dynamics. Equation (43) is, however, well posed as a nonlinear time fractional reaction–diffusion equation that can be derived from a physically consistent CTRW, and thus it should be preferred for testing numerical methods of solution over the single component model equation, Equation (36), obtained by replacing an integer order time derivative with a Caputo fractional order derivative.

## 3. Discussion

Over the past two decades there have been large numbers of papers published on numerical methods for nonlinear fractional reaction–diffusion equations. The original motivation for including time fractional derivatives in reaction–diffusion equations was based on a CTRW description of diffusion with traps and reactions [13]. This description was refined and improved in a series of papers [14,16,17,18,19,20,21,22,23,24], leading to the formulation of time fractional reaction–diffusion equations along the lines of Equation (11). However, many investigations of time fractional reaction–diffusion equations have been carried out on systems obtained by simply replacing integer order time derivatives with Caputo fractional order derivatives. These studies may be interesting from a mathematical analysis point of view but they may not be directly relevant to mathematical modelling applications.

In this paper we have illustrated, through examples, how different time fractional reaction–diffusion equations can be formulated, consistent with an underlying CTRW formalism, taking into account the reaction kinetics. There are three points worth noting in this context: (i) The fractional reaction–diffusion systems considered in this approach are relevant to well-mixed reactions that are not diffusion limited. The reaction dynamics can often be formulated using the law of mass action in these systems. (ii) Different time fractional reaction–diffusion systems can be formulated that are consistent with the same equation for the reaction dynamics. It is important to know the reaction kinetics. (iii) Reaction–subdiffusion equations typically involve a spatial Laplacian operating on a generalized tempered time fractional derivative. The solution of these types of equations would typically require very different numerical approaches than those proposed for reaction–diffusion systems with a fractional order Caputo time derivative replacing the integer order time derivative.

It is hoped that the physically motivated time fractional reaction–diffusion equations, such as Equations (30) and (43), will become more widely used, replacing the simpler ad-hoc equations, such as Equations (33) and (36), as a test for different methods of solution of nonlinear fractional reaction–diffusion systems. Beyond this, there is a real need for physical experiments to be devised and carried out to validate and calibrate time fractional reaction–diffusion models.

## Appendix A. Mean Square Displacements

The mean square displacement of particles evolving according to the fractional diffusion equation

(A1) $$\frac{\partial u(x,t)}{\partial t}=D_{\gamma }\frac{\partial^{2}}{\partial x^{2}}\left[e^{-\alpha t}{\;}_{0}D_{t}^{1-\gamma }\left(e^{\alpha t}u\left(x,t\right)\right)\right],\;\alpha >0.$$

can simply be obtained from the infinite domain Greens function solution G(x,t) with initial condition G(x,0)=δ(x), via

(A2) $$\langle x^{2}\left(t\right)\rangle =\lim_{q\to 0}-\frac{d^{2}}{dq^{2}}\hat{G}\left(q,t\right)$$

where $\hat{G}\left(q,t\right)$ denotes the Fourier transform w.r.t. *x*. We begin by taking the Fourier transform of Equation (A2) and re-arranging terms to write

(A3) $$\frac{\partial }{\partial t}\left(e^{\alpha t}\hat{G}\left(q,t\right)\right)=-q^{2}D_{\gamma }{\;}_{0}D_{t}^{1-\gamma }\left(e^{\alpha t}\hat{G}\left(q,t\right)\right)+\alpha e^{\alpha t}\hat{G}\left(q,t\right).$$

We now introduce

(A4) $$\hat{F}\left(q,t\right)=e^{\alpha t}\hat{G}\left(q,t\right),$$

noting that $\hat{F}\left(q,0\right)=\hat{G}\left(q,0\right)=1$, and then

(A5) $$\langle x^{2}\left(t\right)\rangle =e^{-\alpha t}\lim_{q\to 0}-\frac{d^{2}}{dq^{2}}\hat{F}\left(q,t\right).$$

Starting with the differential equation for $\hat{F}\left(q,t\right)$,

(A6) $$\frac{\partial }{\partial t}\left(\hat{F}\left(q,t\right)\right)=-q^{2}D_{\gamma }{\;}_{0}D_{t}^{1-\gamma }\left(\hat{F}\left(q,t\right)\right)+\alpha \hat{F}\left(q,t\right),$$

we take the Laplace transform w.r.t. time and rearrange terms to write

(A7) $$\hat{\hat{F}}\left(q,s\right)=\frac{1}{\left(s+D_{\gamma }q^{2}s^{1-\gamma }-\alpha \right)}$$

From this we have

(A8) $$\begin{array}{ccc} \lim_{q\to 0}-\frac{d^{2}}{dq^{2}}\hat{\hat{F}}\left(q,s\right) & = & \frac{2D_{\gamma }}{s^{\gamma -1}\alpha^{2}-2s^{\gamma }\alpha +s^{\gamma +1}},\\ \; & = & 2D_{\gamma }\frac{s^{1-\gamma }}{{\left(s-\alpha \right)}^{2}}. \end{array}$$

We now take the inverse Laplace transform using Equation (2.3.26) of [34] to write

(A9) $$\lim_{q\to 0}-\frac{d^{2}}{dq^{2}}\hat{\hat{F}}\left(q,t\right)=2D_{\gamma }t^{\gamma }E_{1,\gamma }^{\left(1\right)}\left(\alpha t\right),$$

and then

(A10) $$\langle x^{2}\left(t\right)\rangle =2D_{\gamma }e^{-\alpha t}t^{\gamma }E_{1,\gamma }^{\left(1\right)}\left(\alpha t\right).$$

## Appendix B. Fox H-Function and Meijer G-Function Solutions

The Fox H-function and the Meijer G-function are defined as path integrals [36]

(A11) $$H_{p,q}^{m,n}\left[z\left|\begin{array}{c} \left(a_{1},A_{1}\right)\left(a_{2},A_{2}\right)\cdots \left(a_{p},A_{p}\right)\\ \left(b_{1},B_{1}\right)\left(b_{2},B_{2}\right)\cdots \left(b_{q},B_{q}\right) \end{array}\right.\right]=\frac{1}{2\pi i}\int_{L}\frac{\prod_{j=1}^{m}\Gamma \left(b_{j}+B_{j}s\right)\prod_{j=1}^{n}\Gamma \left(1-a_{j}-A_{j}s\right)}{\prod_{j=m+1}^{q}\Gamma \left(1-b_{j}-B_{j}s\right)\prod_{j=n+1}^{p}\Gamma \left(a_{j}+A_{j}s\right)}z^{-s}\;ds,$$

and

(A12) $$G_{p,q}^{m,n}\left[z\left|\begin{array}{c} a_{1},a_{2},\cdots a_{p}\\ b_{1},b_{2},\cdots b_{q} \end{array}\right.\right]=\frac{1}{2\pi i}\int_{L}\frac{\prod_{j=1}^{m}\Gamma \left(b_{j}-s\right)\prod_{j=1}^{n}\Gamma \left(1-a_{j}+s\right)}{\prod_{j=m+1}^{q}\Gamma \left(1-b_{j}+s\right)\prod_{j=n+1}^{p}\Gamma \left(a_{j}-s\right)}z^{s}\;ds,$$

respectively, where 0≤n≤p, 1≤m≤q, $\{a_{j},b_{j}\}\in C$, $\left\{\alpha_{j},\beta_{j}\right\}\in R^{+}$, and *L* is a suitably chosen contour. With a simple change of variables it follows that if $A_{j}=C,j=1..p$ and $B_{j}=C,j=1..q$ then

(A13) $$H_{p,q}^{m,n}\left[z\left|\begin{array}{c} \left(a_{1},C\right)\left(a_{2},C\right)\cdots \left(a_{p},C\right)\\ \left(b_{1},C\right)\left(b_{2},C\right)\cdots \left(b_{q},C\right) \end{array}\right.\right]=\frac{1}{C}G_{p,q}^{m,n}\left[z^{\frac{1}{C}}\left|\begin{array}{c} a_{1},a_{2},\cdots a_{p}\\ b_{1},b_{2},\cdots b_{q} \end{array}\right.\right]$$

The Legendre duplication formula

(A14) $$\Gamma \left(2z\right)=\frac{2^{2z-1}}{\sqrt{\pi }}\Gamma \left(z\right)\Gamma \left(z+\frac{1}{2}\right)$$

is useful for reducing Fox H-functions to Meijer G-functions in the expressions below.

The Fox-H function

(A15) $$H_{1,2}^{2,0}\left[z\left|\begin{array}{c} \left(1-\frac{\gamma }{2}+j,\gamma \right)\\ \left(0,1\right)\left(\frac{1}{2}+j+1\right) \end{array}\right.\right]=\frac{1}{2\pi i}\int_{L}\frac{\Gamma \left(s\right)\Gamma \left(\frac{1}{2}+j+s\right)}{\Gamma (1-\frac{\gamma }{2}+j+\gamma s)}z^{-s}\;ds$$

appears in the solutions, Equations (21) and (25). Here we show how, in the case $\gamma =\frac{1}{2}$, this can be represented as a Meijer G-function leading to the solutions in Equations (26) and (27). With $\gamma \;=\;\frac{1}{2}$ in Equation (A15) we have

(A16) $$H_{1,2}^{2,0}\left[z\left|\begin{array}{c} \left(\frac{3}{4}+j,\frac{1}{2}\right)\\ \left(0,1\right)\left(\frac{1}{2}+j+1\right) \end{array}\right.\right]=\frac{1}{2\pi i}\int_{L}\frac{\Gamma \left(s\right)\Gamma \left(\frac{1}{2}+j+s\right)}{\Gamma (\frac{3}{4}+j+\frac{s}{2})}z^{-s}\;ds.$$

We now use the duplication formula, Equation (A14), to replace

(A17) $$\Gamma \left(s\right)=\frac{2^{s-1}}{\sqrt{\pi }}\Gamma \left(\frac{s}{2}\right)\Gamma \left(\frac{s}{2}+\frac{1}{2}\right),$$

and

(A18) $$\Gamma \left(\frac{1}{2}+j+s\right)=\frac{2^{\frac{1}{2}+j+s-1}}{\sqrt{\pi }}\Gamma \left(\frac{1}{4}+\frac{j}{2}+\frac{s}{2}\right)\Gamma \left(\frac{3}{4}+\frac{j}{2}+\frac{s}{2}\right),$$

so that

(A19) $$\begin{array}{ccc} H_{1,2}^{2,0}\left[z\left|\begin{array}{c} \left(\frac{3}{4}+j,\frac{1}{2}\right)\\ \left(0,1\right)\left(\frac{1}{2}+j+1\right) \end{array}\right.\right]\; & = & \frac{1}{2\pi i}\int_{L}\frac{2^{\frac{1}{2}+j+2s-2}}{\pi }\frac{\Gamma \left(\frac{s}{2}\right)\Gamma \left(\frac{1}{2}+\frac{s}{2}\right)\Gamma \left(\frac{1}{4}+\frac{j}{2}+\frac{s}{2}\right)\Gamma \left(\frac{3}{4}+\frac{j}{2}+\frac{s}{2}\right)}{\Gamma (\frac{3}{4}+j+\frac{s}{2})}z^{-s}\;ds,\\ \; & = & \frac{2^{\frac{1}{2}+j-2}}{\pi }H_{1,4}^{4,0}\left[\frac{z}{4}\left|\begin{array}{c} \left(\frac{3}{4}+j,\frac{1}{2}\right)\\ \left(0,\frac{1}{2}\right)\left(\frac{1}{2},\frac{1}{2}\right)\left(\frac{1}{4}+\frac{j}{2},\frac{1}{2}\right)\left(\frac{3}{4}+\frac{j}{2},\frac{1}{2}\right) \end{array}\right.\right]\\ \; & = & \frac{2^{j}}{\sqrt{2}\pi }G_{1,4}^{4,0}\left[{\left(\frac{z}{4}\right)}^{2}\left|\begin{array}{c} \frac{3}{4}+j\\ 0,\frac{1}{2},\frac{1}{4}+\frac{j}{2},\frac{3}{4}+\frac{j}{2} \end{array}\right.\right]. \end{array}$$

Note that if j=0 this simplifies further to

(A20) $$\frac{1}{\sqrt{2}\pi }G_{1,4}^{4,0}\left[{\left(\frac{z}{4}\right)}^{2}\left|\begin{array}{c} \frac{3}{4}\\ 0,\frac{1}{2},\frac{1}{4},\frac{3}{4} \end{array}\right.\right]=\frac{1}{\sqrt{2}\pi }G_{0,3}^{3,0}\left[{\left(\frac{z}{4}\right)}^{2}\left|\begin{array}{c} -\\ 0,\frac{1}{2},\frac{1}{4} \end{array}\right.\right].$$

The Meijer G-functions above are of the general form $G_{p,q}^{q,0}\left(z\right)$ where asymptotic expansions are known for z≫1 [37]. In particular, using Equation (22) in [37], we have

(A21) $$G_{1,4}^{4,0}\left[z\left|\begin{array}{c} \frac{3}{4}+j\\ 0,\frac{1}{2},\frac{1}{4}+\frac{j}{2},\frac{3}{4}+\frac{j}{2} \end{array}\right.\right]∼\exp\left(-3z^{\frac{1}{3}}\right)z^{-\frac{1}{12}}\sum_{k=0}^{\infty }\frac{M_{k}\left(j\right)}{z^{\frac{k}{3}}}$$

for all j∈N and z≫1, where the $M_{k}\left(j\right)$ are functions of the parameters, including *j*, but not the variable *z*. If we let *M* denote the largest $M_{k}\left(j\right)$ then we can evaluate the sum as a geometric series to write

(A22) $$G_{1,4}^{4,0}\left[z\left|\begin{array}{c} \frac{3}{4}+j\\ 0,\frac{1}{2},\frac{1}{4}+\frac{j}{2},\frac{3}{4}+\frac{j}{2} \end{array}\right.\right]∼M\frac{\exp\left(-3z^{\frac{1}{3}}\right)z^{\frac{1}{4}}}{z^{\frac{1}{3}}-1}$$

and similarly for $G_{0,3}^{3,0}$.
